# Porous Deep Eutectic Solvents–Unfulfilled Dream or the Next Breakthrough in Scientific Innovation?

**DOI:** 10.1002/advs.202412622

**Published:** 2024-12-24

**Authors:** Marcin Wysokowski, Patrycja Makoś‐Chełstowska, Alina Brzęczek‐Szafran, Aleksandra Sikora, Adam Gorczyński, Teofil Jesionowski

**Affiliations:** ^1^ Faculty of Chemical Technology Institute of Chemical Technology and Engineering Poznan University of Technology Berdychowo 4 Poznan 60965 Poland; ^2^ Department of Process Engineering and Chemical Technology Faculty of Chemistry Gdansk University of Technology Gdańsk 80‐233 Poland; ^3^ Department of Chemical Organic Technology and Petrochemistry Silesian University of Technology Gliwice 44‐100 Poland; ^4^ Faculty of Chemistry Adam Mickiewicz University Uniwersytetu Poznańskiego 8 Poznań 61‐614 Poland

**Keywords:** absorption, deep eutectic solvents, green chemistry, sustainable porous materials

## Abstract

Porous deep eutectic solvents (PDES) are capturing the imagination of scientists, promising a revolutionary leap in material science. These innovative materials, blending the versatility of deep eutectic solvents (DES) with the intricate architectures of porous structures, offer an exciting array of applications—from green chemistry and catalysis to energy storage and environmental remediation. However, the journey from laboratory curiosity to industrial application is fraught with challenges. This perspective article analyzes the realm of PDES, scrutinizing the cutting‐edge advancements and the challenges that lie ahead. By exploring their synthesis, unique properties, and diverse application potential, the critical question is asked: are PDES an unfulfilled dream or the next big breakthrough in scientific innovation? A comprehensive analysis reveals a “landscape” ripe with opportunity, suggesting that with targeted research and development, PDES can indeed become a cornerstone technology, driving progress across multiple scientific domains.

## Graphical abstract

This perspective examines the potential of porous deep eutectic solvents (PDES) in gas absorption, separation, and environmental applications. Highlighting their advantages in sustainability, low toxicity, and ease of synthesis, the article discusses the challenges and future directions of PDES in advancing green chemistry and industrial processes.

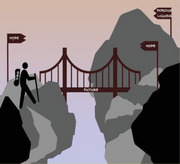

## 1 Introduction

Porous materials, characterized by regions of empty space, offer the unique capability to selectively adsorb guest molecules, facilitating their chemical transformation.^[^ ^1^ ^]^ This intrinsic property has rendered them invaluable across a spectrum of applications, including gas separation, energy storage, ion exchange, heterogeneous catalysis, and green chemistry.^[^ ^2^ ^]^ Traditionally, these materials are ordered (amorphous or crystalline) solids, providing a structured framework that supports their functional versatility in both industrial and domestic settings.^[^ ^3^ ^]^ Recently, the advent of porous liquids, first proposed in 2007,^[^ ^4^ ^]^ has introduced a new dimension to this field, combining the fluidity of liquids with the advantageous size and shape selectivity of porous domains.^[^ ^5^ ^]^ This innovative class of materials promises to expand the utility of porous systems, opening new avenues for research and application.^[^ ^6^ ^]^

Porous liquids (PLs) benefit from the integration of the following features:

(i) superfluidity that results in rapid heat dissipation, which is an advantage to the absorption and regeneration profiles. Meanwhile, the advantage of excellent processable liquid provides several engineering techniques to tackle the problem of slow gas diffusion in liquids, including agitation, spraying, membrane contactors, etc;
(ii) elimination of the daunting issues of solid sorbent materials (i.e., physical aging, plasticization, etc.);
(iii) can be used in industrial pumps due to superior flowing ability;
(iv) well‐defined construction of porous solids materials, provides cavities to accumulate other molecules. Moreover, the size and volume of these cavities can be designed for specific usage.

**Figure 1:**
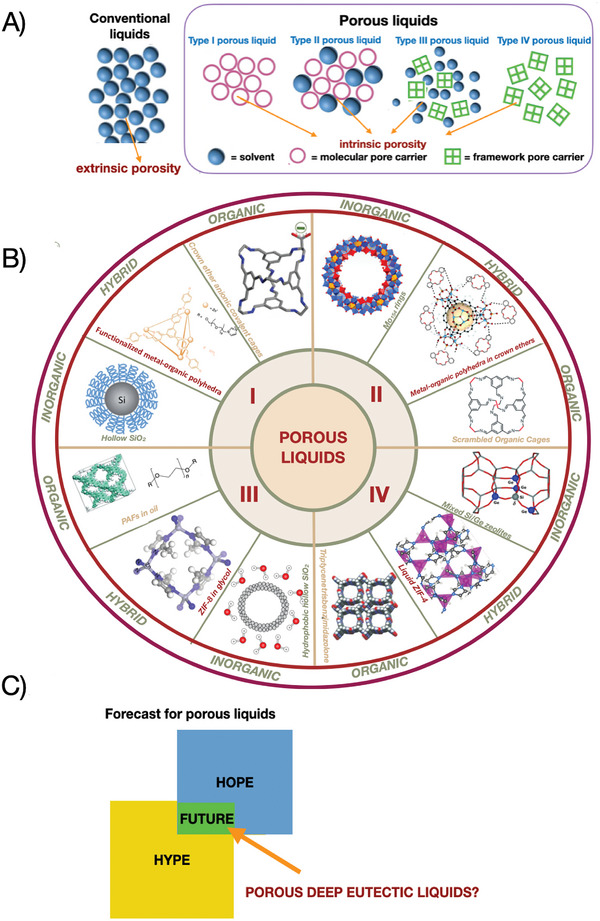
A) Classification of porous liquids; B) Examples of different types of porous liquids with corresponding structures (adapted with permission from John Wiley and Sons;^[^ ^10^ ^]^ Springer Nature Ltd.^[^ ^8^, ^11^, ^12^, ^13^ ^]^) C) Future forecast for porous liquids, emphasizing the potential of PDES as a game‐changer in the field.

Porous liquids are divided into three main types; however, some sources report four types of them.^[^ ^7^, ^8^ ^]^ The feature that unites all types of PLs is the absence of the self‐adsorption phenomenon, implying that a PL retains continuously open and accessible pores.^[^ ^6^ ^]^ Type I porous liquids, the most challenging to synthesize, are neat liquid materials in which the individual molecules possess permanent, rigid internal cavities. Type II porous liquids consist of rigid hosts dissolved in solvents which are sterically prevented from entering the host cavities. Type III liquids are dispersions of microporous frameworks in solvents that are sterically prevented from entering the pores. Type IV–they are solid materials of extended connectivity in three dimensions, which, in the liquid state, retain intrinsic porosity due to the strong constraints of their instantaneous coordination: that is, the local order imposed by the associated nature of the liquid.^[^ ^9^ ^]^ All types are schematically represented in **Figure** **1**a. As we do not intend here to review the present state of art regarding porous liquids, for better understanding we strongly recommend the following articles.^[^ ^5^, ^6^, ^7^, ^8^, ^9^ ^]^

Following the successful proof‐of‐concept and synthesis of porous liquids, scientific attention has now shifted toward their sustainability. Researchers are increasingly focusing on creating porous liquids that are not only effective but also environmentally friendly, utilizing green chemistry principles to minimize their ecological footprint. Most of the scientific attention is shifted toward the development of porous ionic liquids.^[^ ^14^, ^15^, ^16^ ^]^ The utilization of ionic liquids in the formation of porous liquids has several advantages, not least the negligible vapor pressure of most systems. Ionic liquids were incorporated into type I, II, and type III systems. The preparation of type I or type II porous liquids demands considerable effort, whereas type III porous liquids, which are gaining increased interest, can be easily produced by dispersing porous solids in the appropriate solvents.

Pioneering works by Costa Gomes et al. revealed the potential of type III systems consisting of simply mixed, commercially available zeolitic imidazolate frameworks ZIF‐8 and the trihexyltetradecylphosphonium bis(trifluoromethylsulfonyl) imide ionic liquid [P6,6,6,14][NTf_2_] in the reversible absorption of gases. The suspension absorbed 60% more CO_2_ and over 100% more methane or nitrogen than the corresponding neat ionic liquid and the gas absorption was proportional to the amount of porous solid in the suspension.^[^ ^14^, ^15^ ^]^ As indicated by the authors, ionic liquids with intrinsic Coulombic interactions, offer a considerable advantage in the design of porous liquids compared to molecular solvents due to cohesive energy that prevents small ions from entering the pores. Therefore, there is no need to design an ionic liquid with both ions larger than the cavities. By taking advantage of Coulombic interactions, as well as the electrostatic interactions and hydrogen bonding that ionic liquids can generate with a porous host, various voluminous ionic systems have been designed and synthesized for the preparation of different types of porous liquids. The group of Dai successfully liquefied porous carbons by anchoring to the surface of hollow carbon spheres polymerized imidazolium‐based ionic liquids with polyethylene glycol (PEG) incorporated into the sulfonate anions.^[^ ^17^ ^]^ The combination of both systems into type I porous liquid was possible via *π*–*π* interactions between the imidazolium cations and the carbon networks. The same group also proposed the incorporation of PEG into the DBU‐based cation, forming a bulky ionic liquid that provided permanent porosity to type III porous liquids formed with ZIF‐8, ZSM‐5, and Silicalite‐1.^[^ ^18^ ^]^ Bulky ionic liquid with PEG incorporated into the bisimidazolium cation was also reported by Dinker et al. who synthesized an ionic liquid with a molecular size larger than the pore aperture of metal–organic polyhedra (Pd_12_L_24_) used as a porous host for type II porous ionic liquids.^[^ ^19^ ^]^ Another approach was presented by Nitschke and co‐workers who developed type I porous liquids by functionalizing a coordination cage with PEG chains terminated with imidazolium cations whose role was keeping the chains from entering the positively charged cage cavity.^[^ ^11^ ^]^

Depending on their design, porous ionic liquids demonstrate immense potential for absorbing large quantities of various gases and selectively separating them. Solvents applied for the construction of PILs can facilitate or prevent the diffusion of gas into the porous solid, depending on the solubility of gas molecules.

Nevertheless, this emerging field still faces several challenges and obstacles that limit their practical utilization. Economic as well ecologic aspects of the synthesis of chemically and thermally robust porous liquids, as well as their scaling up are the main limiting factors. Most of the examples prepared to date are just prototypes prepared on a small scale, using multistep synthesis protocols and toxic solvents, despite significant progress in the design of easy‐to‐prepare type III porous liquids. Therefore, the challenges for the future include i) reducing their cost by further simplifying the architecture and components of porous liquids and ii) improving sustainability.

To address these challenges, researchers are increasingly looking towards alternatives that combine sustainability with high performance and deep eutectic solvents (DES), known for their low toxicity, biodegradability, and cost‐effective preparation method, have emerged as a promising sustainable alternative to traditional ionic liquids.^[^ ^20^, ^21^, ^22^, ^23^ ^]^

## 2 Porous Deep Eutectic Solvents (PDES)

As the quest for more sustainable porous materials continues, deep eutectic solvents (DES) offer a compelling alternative due to their inherent environmental benefits and cost‐effectiveness. By integrating DES with porous structures, researchers can potentially create new classes of porous liquids that retain the desirable properties of traditional porous materials while leveraging the ecological advantages of DES. This innovative approach could not only address the sustainability issues associated with conventional ionic liquids but also open up new possibilities for the practical application of porous liquids in various fields. Surprisingly, DESs that are considered as alternatives to ILs, are still neglected/poorly investigated in the development of porous liquids. It is thought‐provoking, but definitively it is worth investigating due to the sustainability and the properties that it can offer. The recent advances demonstrated that DES‐amended MOFs can unlock efficient toxic gases capture paths thanks to MOFs' favorable features for gas separation such as tunable porosity. We are convinced that a rational combination of metal–organic frameworks (MOFs), covalent organic frameworks (COFs) as well as H‐bonded organic frameworks (HOFs) and supramolecular organic frameworks (SOFs) with DESs will also greatly enhance low‐pressure absorption of toxic gases, correspondingly as it has been achieved already for ILs.^[^ ^23^, ^24^ ^]^ Compared with ILs, DESs have lower toxicity, greater biodegradability, lower price, and much simpler and waste‐free synthesis. With no doubts development of PDES will be game‐changing in terms of the development of sustainable, high‐performance technologies for air purification and separation of gases.^[^ ^25^ ^]^ Apparently, type III deep eutectic liquids seem the most facile type to prepare. On the other hand, MOFs and COFs can be endowed with H‐bonding donors and acceptors. This is still an open question if this strategy can be used to actually prevent the migration of components of DES into the MOF/COF framework. (E.g., MOF with HBA can bind some of the HBD, which would normally be part of HBA/HBD DES system).

To be beneficial in the absorption process, a PDES must fulfill specific criteria. The primary requirement is to maintain the porosity of the liquid by preventing DES components from entering the pores of the MOFs or COFs. For this purpose, the properties of MOF and COF can be helpful. Their structures can be freely modified, especially by acting within their side groups. If the modification is designed in the right way, it is possible to obtain groups equipped with hydrogen bond donors or acceptors (HBD/HBA), which will serve to control the migration of DES components into the MOF/COF structure. The bonds formed during the synthesis or dissolution of such materials in DES can stabilize the solvent components on the surface, preventing their migration into the organic structure. Unlike dispersing pristine MOFs in ILs, which can result in highly viscous systems and reduced process efficiency, the proposed modification approach ensures a better balance between porosity and fluidity. Furthermore, the strong hydrogen bonding of DES with functionalized MOF surfaces provides superior control over solvent interactions, which is not feasible in IL‐based systems where solvent penetration is less problematic but compromises on tunability and eco‐friendliness. This strategy thus offers not only the structural and functional advantages of MOFs but also leverages the sustainable and cost‐effective nature of DES. This is a promising strategy that can be used to solve a variety of problems, including separation, catalysis, and energy storage, where the stability of DES components is crucial.

On the other hand, the concept of dispersing COFs in DES mixtures necessitates a different approach, i.e., different mixtures and molar ratios than those that are typically used. Blocking the pores restricts access of contaminants to the pores, thereby minimizing their removal efficiency. Ionic liquids possess the unique characteristic of maintaining porosity, even in the presence of small anions, as a result of strong Coulomb interactions between cations and anions, which leads to cohesion energy that prevents the separation of cations and anions.^[^ ^26^ ^]^ In DES, the organic compounds can be small enough to gain access to the pores, thereby reducing the porosity of the suspensions. Although DES maintains a high cohesion energy owing to hydrogen bonds, it is not clear whether this is sufficient to keep the molecular compound out of the pores. Further research is required to clarify this phenomenon. Another solution is to use a DES composed of components with large structures, whose sizes are larger than the pore diameters. However, using large‐molecule components to obtain DES can be a challenge in receiving liquid PDES owing to the high melting points of the individual components.

**Figure 2:**
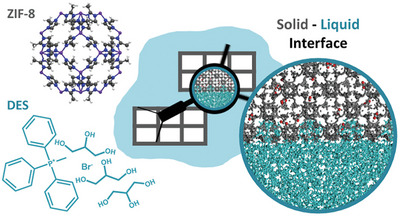
Chemical structures of the components of the porous DES. Methyltriphenylphosphonium bromide with glycerol in a molar ratio of 1:3 ([MePh_3_P][Br]/Gly) and ZIF‐8. Together with MD simulations of DES‐MOF interactions on solid–liquid interface. Reprinted with permission from.^[^ ^26^ ^]^ Copyright 2024 American Chemical Society.

Pioneering work by Li et al. proved that it is possible to incorporate DES into UiO‐66 by preserving the open metal sites for CO_2_ capture.^[^ ^27^ ^]^ However, the resulting material was not in liquid form, despite the use of components with relatively small structures, that is, choline chloride and urea. Recently, the groups of Padua and Costa Gomes formed a stable suspension by combining a DES consisting of a voluminous phosphonium salt (methyltriphenylphosphonium bromide) and glycerol with ZIF‐8, where the size of glycerol (3.6 Å) is comparable to the apertures in ZIF‐8 (3.4 Å).^[^ ^23^, ^28^ ^]^ Stable up to 5 months suspension consisting of both the solid particles and the liquid, as evidenced by X‐ray scattering. Supported by MD simulations the authors confirmed that DES does not fill the pores of MOF but accommodates in the surface‐accessible cavities of ZIF‐8.^[^ ^26^ ^]^ Thus, the origin of porosity might not solely be attributed to the Coulomb interaction between cations and anions, or the cohesive energy that prevents small ions from entering the pores, as seen in porous ILs. Instead, in these systems, the significant hydrophobicity of ZIF‐8 or the challenge of opening the ZIF‐8 apertures at moderate pressure is believed to contribute to the formation of a porous suspension using only glycerol. Experimental gas absorption tests confirmed the porosity of these suspensions in glycerol, showing a notable increase in CO_2_ absorption compared to pure glycerol, indicating that the pores in the particles serve as gas reservoirs, consistent with the simulations.^[^ ^29^ ^]^ (**Figure** **2**).

Another potential problem could also be the viscosity of PDES, as high viscosity slows gas diffusion through the gas–liquid interface and consequently adversely affects the mass exchange process. On the other hand, an increase in the size of the molecules used to obtain DES increases their viscosity.^[^ ^23^, ^30^ ^]^ These problems could be solved by simply adding PEG chains to the system. The long PEG chains not only protect the host cavities by making them accessible (for gas molecules), but also lower the melting point of the porous metal‐organic cage, and cause it to behave as a liquid. He et al.^[^ ^30^ ^]^ report that the self‐assembly of long polyethylene glycol (PEG)‐imidazolium chain‐functionalized linkers, calixarene molecules, and Zn ions results in the formation of a porous liquid. This material not only enhances the performance of CO_2_ capture but also outperforms non‐modified solid metal‐organic cages. This strategy shows great promise for the formation of porous deep eutectic solvents (PDES) for the capture and neutralization of toxic gases.

Next worth considering the potential application of PDES covers photoswitches, which are compounds that exhibit reversible structural changes upon light irradiation. Examples include azoarenes, stilbenes, spiropyrans, and dithienylethenes. In designing a photoresponsive PL inspiration for these new materials can be taken from many different areas, where elements of learning from both photoswitch and porous material design could be combined to develop new strategies towards new photoresponsive PLs.^[^ ^31^ ^]^ Despite their potential, the field of photoresponsive porous liquids remains underexplored.^[^ ^32^, ^33^ ^]^ This study may inspire the development of a new adsorption process regulated by light instead of pressure and temperature swing adsorption, as Dinker et al have done in their work.^[^ ^34^ ^]^However, photoresponsivity is not the only way to use these materials. Another possible application covers the use of PDES in gas storage systems. Poly(dimethylsiloxane) (PDMS) is an attractive material for PL gas storage and transport for several reasons: PDMS is inexpensive, nontoxic, and has a low vapor pressure, high thermal stability, and good H_2_ solubility. The fluidity of PDMS down to −40 °C also makes the solvent promising for low‐viscosity PL applications. While linear PDMS cannot be easily grown as a robust coating around framework‐based colloids using conventional polymerization techniques, in principle, any vinyl monomer compatible with ATRP could be employed for the polymeric coating in such materials.^[^ ^35^ ^]^

Furthermore, the PDES must have a high affinity for impurities to be removed and a low affinity for the gas matrix. In addition, we can't forget about the properties dedicated to green solvents, i.e., low vapor pressure, no or low toxicity, and high biodegradability. Meeting all the requirements is challenging, but the potential benefits of obtaining porous DES capable of gas separation are enormous. Further research should synergically involve theoretical physical chemists, synthetic organic chemists, material scientists as well as modern deep learning and machine learning techniques^[^ ^7^, ^36^ ^]^ that with the assistance of AI will be able to predictively design such porous deep eutectic solvents in the future.

## 3 Conclusion and Future Outlook

Porous deep eutectic solvents (PDES) represent a burgeoning scientific frontier, poised to bridge the gap between sustainability and high‐performance applications in green chemistry, catalysis, gas separation, and energy storage. While PDES harness the benefits of deep eutectic solvents (DES)—such as low toxicity, biodegradability, and cost‐effectiveness—they also leverage the intricate porosity of advanced materials like metal‐organic frameworks (MOFs) and covalent organic frameworks (COFs). Despite their potential, the development of PDES remains in its infancy, with many challenges yet to be overcome. These include preventing the migration of DES components into porous frameworks, managing high viscosities, and scaling up sustainable synthesis protocols. Nevertheless, the immense promise of PDES as a sustainable alternative to conventional porous liquids makes it a compelling area for further investigation. Future work should focus on optimizing these solvents for large‐scale industrial processes while maintaining their green chemistry principles—low toxicity, high biodegradability, and low environmental impact. Additionally, advancements in sustainability criteria may involve the design of porous liquids based on components with biorenewable origins, as demonstrated with glycerol‐based DES.^[^ ^23^ ^]^ The increased interest in the design of sustainable solvents applies to both DES as well as ionic liquids that can be prepared using precursors sourced from renewable sources such as vegetable or algae‐derived oils, cellulose, chitin, starch, and proteins.^[^ ^37^ ^]^ Many biomass‐derived compounds possess structural features (functional groups, aromatic moieties) that can favor interactions with porous hosts through H‐bonding or *π*–*π* interactions.

Beyond gas separation and storage, PDES could find uses in novel areas such as light‐responsive materials (photoswitches) and advanced adsorption processes. Further exploration of these applications could open up new scientific niches for PDES. We are more than convinced that with targeted research into overcoming current challenges, PDES could evolve from a promising concept into a practical, sustainable solution for diverse industrial applications, aligning with global efforts toward greener technologies.

## Conflict of Interest

The authors declare no conflict of interest.
